# Theory of Mind Development in Adolescence and Early Adulthood: The Growing Complexity of Recursive Thinking Ability

**DOI:** 10.5964/ejop.v11i1.829

**Published:** 2015-02-27

**Authors:** Annalisa Valle, Davide Massaro, Ilaria Castelli, Antonella Marchetti

**Affiliations:** a Department of Psychology, Università Cattolica del Sacro Cuore, Milan, Italy; University College Cork, Cork, Ireland

**Keywords:** recursive thinking, theory of mind, adolescence, adulthood, third-order false-belief task

## Abstract

This study explores the development of theory of mind, operationalized as recursive thinking ability, from adolescence to early adulthood (*N* = 110; young adolescents = 47; adolescents = 43; young adults = 20). The construct of theory of mind has been operationalized in two different ways: as the ability to recognize the correct mental state of a character, and as the ability to attribute the correct mental state in order to predict the character’s behaviour. The Imposing Memory Task, with five recursive thinking levels, and a third-order false-belief task with three recursive thinking levels (devised for this study) have been used. The relationship among working memory, executive functions, and linguistic skills are also analysed. Results show that subjects exhibit less understanding of elevated recursive thinking levels (third, fourth, and fifth) compared to the first and second levels. Working memory is correlated with total recursive thinking, whereas performance on the linguistic comprehension task is related to third level recursive thinking in both theory of mind tasks. An effect of age on third-order false-belief task performance was also found. A key finding of the present study is that the third-order false-belief task shows significant age differences in the application of recursive thinking that involves the prediction of others’ behaviour. In contrast, such an age effect is not observed in the Imposing Memory Task. These results may support the extension of the investigation of the third order false belief after childhood.

## Introduction

Theory of mind is the ability to understand mental states (intentions, emotions, desires, beliefs), and to predict one’s own and others’ behaviour on the basis of these states (Premack & Woodruff, 1978). Theory of mind research has traditionally been conducted in children and has identified the key moments of its development using false belief tasks. The first-order false-belief task evaluates recursive thinking of the first level (i.e. ‘I think that you think’), which appears around 4 years of age (Wimmer & Perner, 1983); the second-order false-belief task measures recursive thinking of the second level (i.e. ‘I think that you think that she/he thinks’), which is evident at around 8 years of age (Perner & Wimmer, 1985). In recent years, there has been increasing interest in theory of mind abilities beyond childhood, which are investigated through the use of a wider and more variable set of tasks that differs from the false-belief task (Henry, Phillips, Ruffman, & Bailey, 2013; Miller, 2012). Indeed, studies of non-infant subjects show that theory of mind is often related to other significant social competences (Filippova & Astington, 2010; Massaro, Valle, & Marchetti, 2013; Massaro, Valle, & Marchetti, 2014) and that it evolves also after infancy and childhood (Apperly, Samson, & Humphreys, 2009; Dumontheil, Apperly, & Blakemore, 2010; Sommerville, Bernstein, & Meltzoff, 2013). A particularly interesting period is adolescence, since it is characterized by major changes in the cognitive, socio-emotional, and relational domains (Eccles, Templeton, Barber, & Sotone, 2003). Moreover, the increasing importance of interpersonal relationships in adolescence (Kenny, Dooley, & Fitzgerald, 2013), especially among peers, requires the frequent and accurate use of specific social skills, such as the ability to understand one’s own and others' minds.

The study of theory of mind in adolescence and early adulthood constitutes a methodological challenge, because it requires the creation of new theory of mind tasks in order to capture age differences (Henry et al., 2013; Moran, 2013). Despite the fact that the literature has provided a discrete number of tasks to test theory of mind after childhood, it may be interesting to notice that the construct of false belief, so relevant to study theory of mind in infancy and childhood, has been mostly neglected and replaced by other measures which evaluate mainly social and emotional-affective aspects of theory of mind.

As regards the social aspects of theory of mind, the tasks that have been created consist of stories or cartoons about various types of social situations, where subjects have to detect the reasons for a character’s behaviour. For example, in the Strange Stories (Happé, 1994), the subject has to provide a mentalistic explanation of the behaviour of a character in situations of misunderstanding, white lie, irony, persuasion and so on. In the Faux Pas Task (Stone, Baron-Cohen, & Knight, 1998) the subject has to recognize the presence of a “gaffe” in a social interaction. Finally, in the Social Understanding Tasks devised by Bosacki and Astington (1999) the subject has to answer some questions regarding a social situation, referring to conceptual role-taking, empathetic sensitivity, person perception, and so on. Similar measures to the Strange Stories and the Faux Pas Task are those devised by Vetter, Leipold, Kliegel, Philips, and Altgassen (2013) and Altgassen, Vetter, Phillips, Akgün, and Kliegel (2014).

As regards the emotional-affective aspects of theory of mind, a widely used task is the Reading the Mind in the Eyes Test (Baron-Cohen, Wheelwright, Hill, Raste, & Plumb, 2001), where the subject has to choose the best label for the description of the emotional mental states from a character’s eye gaze. Other tasks evaluate the ability of perspective-taking (see for example Choudhury, Blakemore, & Charman, 2006), trying to distinguish between a cognitive and an affective component of theory of mind: this is the case of the perspective-taking task devised by Shamay-Tsoory, Harari, Aharon-Peretz, and Levkovitz (2010) and of the cartoons used by Sebastian et al. (2012).

Referring to the more broad construct of mentalization, different types of measures are used, such as the Mentalising Stories for Adolescence (Vrouva & Fonagy, 2009), the Social Cognition and Object Relation Scale – SCORS (Westen, Lohr, Silk, Gold, & Kerber, 1990), in particular the two sub-scales about mentalizing (Rothschild-Yakar, Levy-Shiff, Fridman-Balaban, Gur, & Stein, 2010), the Levels of Emotional Awareness Scale for Adolescents ALEAS (Pratt, 2006), the MASC Movie for the Assessment of Social Cognition (Dziobek et al., 2006).

It may be interesting to underline that in the general overview provided so far, the tasks evaluate the socio-relational components of theory of mind, that belong to the implicit aspects of theory of mind reasoning (Ruffman, 2014), thus neglecting the evaluation of the increasing complexity of meta-representational recursivity. In our view, the only tasks that try to evaluate the recursive thinking in adolescents and young adults, are those devised by Dumontheil et al. (2010) and by Bernstein, Thornton, and Sommerville (2011). In the first case, participants observed a series of objects put on a set of shelves: they had to move the objects following the instructions of a “director of communication”, knowing that the latter could see only some of the objects. The subjects have to act on the basis of their ability to disentangle their own knowledge of the reality and the director’s knowledge, a cognitive operation similar to the one involved in the resolution of the false-belief task. In the second case, Bernstein et al. (2011) proposed a continuous false-belief task that differs from the classic dichotomous false-belief task. In this new task, the subject is required to predict where a character in the story will look for a target that was moved to a different location inside the same box. This task is the only one that measures the false-belief understanding, with a new method of answer that allows a continuous and more detailed measurement.

We think that it is worth continuing to study the understanding of false-belief beyond childhood and to create new tasks for this purpose for two reasons. First, although in the history of theory of mind research, it has been conceived as the “litmus-paper” of the meta-representational ability, there is a lack of knowledge about its development after childhood. Second, the false-belief task evaluates the pure cognitive mechanism that underlies theory of mind reasoning, which may not be fully caught by the social and emotional-affective tasks. In these last tasks, people may answer correctly mainly thanks to other strategies, such as the reference to a familiar social context, or to heuristics (Keysar, Lin, & Barr, 2003; Massaro, Castelli, 2009).

In order to outline the development of the cognitive dimension of theory of mind till early adulthood, we analyse different levels of recursive thinking. We developed and contributed to the validation of a third-order false-belief task following the classical structure of the unexpected transfer task (Wimmer & Perner, 1983) increasing the meta-representational level of recursivity until the third embedded belief (‘I think that you think that he/she thinks that another person thinks…’). This ability has been explored along with the evaluation of general cognitive abilities. In fact, there is consistent evidence that theory of mind development, from its onset to its decline, is related to cognitive abilities such as language, memory and executive function (Apperly et al., 2009; German & Hehman, 2006; Mutter, Alcorn, & Welsh, 2006). Thanks to their more sophisticated cognitive abilities, adults are generally faster and more accurate than children when completing both simple and complex experimental tasks. However, even adults show some limitations in responding to theory of mind tasks (Epley, Keysar, Van Boven, & Gilovich, 2004; Keysar et al., 2003). This seems to support the hypothesis that complex tasks require substantial cognitive engagement, which interferes with theory of mind reasoning (Apperly et al., 2009). However, it is also interesting to notice that, in their examination of theory of mind in people aged 7 to 27, Dumontheil et al. (2010) found that perspective-taking ability continues to improve in late adolescence, even if executive function and working memory have already reached adult levels.

### Aims and Hypotheses

Given the evidence we have discussed that theory of mind and corresponding recursive thinking ability continue to develop after childhood, this study explores these abilities in subjects aged 14, 17, and 20 years. In addition, this study proposes a contribution to the validation of a new third-order false-belief task (structured as an unexpected transfer task).

We aim to explore the development of two types of recursive thinking and their possible link from adolescence to adulthood. In the first type, the subject must interpret the thoughts of the characters (the Imposing Memory Task; Kinderman, Dunbar, & Bentall, 1998). The second requires the subject to predict the character’s behaviour on the basis of her/his mental state (the new third-order false-belief task). Each of the two types of recursive thinking is in turn articulated in different levels of increasing complexity. So, they measure the same construct (theory of mind) but with different operationalizations: in the first case the subject has to recognize the correct mental state of the character; in the second case the subject has to attribute the correct mental state in order to predict the character’s behaviour. We also assess the relationships among working memory, language ability, and executive function and the two types of recursive thinking. We hypothesize that the two types of recursive thinking correlate at least when they evaluate the same level of meta-representation; we predict that the role of these cognitive abilities will be more prominent in the more complex recursive thinking. We also hypothesize a stronger age effect on behaviour prediction than on mental state recognition after controlling cognitive abilities, showing a greater complexity of the former capacity.

## Method

### Participants

One hundred and ten subjects participated. Of these, 47 were 14-year-olds (young adolescents; *M* = 14.8 years, *SD* = 0.49 years, females = 22), 43 were 17-year-olds (adolescents; *M* = 17.8 years, *SD* = 0.33 years, females = 18), and 20 were 20-year-olds (young adults; *M* = 22.8 years, *SD* = 1.7 years, females = 10). All participants were Italian. The two groups of adolescents were recruited in high schools, and the young adults were recruited from the University. They were neither referred to social services, nor reported for learning and socio-relational difficulties. Informed consent was obtained from each participant, and informed parental consent was obtained for the two groups of adolescents. The research was conducted according to APA ethical standards and was approved by the local ethics committee.

### Materials and Procedure

#### Theory of Mind Tasks

Recursive thinking ability was assessed by two tasks: the Imposing Memory Task and a third-order false-belief task, which was specifically devised for the present research.

The Imposing Memory Task (IMT; Kinderman et al., 1998) is an advance theory of mind task composed of a series of five stories: four mentalistic stories that describe a complex social situation, and one control story that involves one character. Each mentalistic story requires the subject to apply recursive thinking to understand the perspective and the intentions of the characters: the subject is required to answer a questionnaire about the characters’ mental states (recursive thinking questions) or about information in the story (memory questions). The response format for each question is a forced choice between two alternatives: one correct and one incorrect. The questions collectively assess different levels of recursive thinking, ranging from the first level of complexity (about a character’s mental state; for example ‘Sam wanted to go to the Post Office to buy a stamp/a tax disc’) to the fifth level of complexity (involving different characters’ minds; for example ‘John thought that Pamela thought that he, John, wanted that Pamela discovered what Sara wanted to do because John wanted to go out alone with Pamela’). Since not all stories include questions that assess the fifth level of complexity, we calculated one score for each level of recursive thinking and proportioned these scores (the range is 0–1). The range of the total score, obtained by the sum of the score of all levels, was similarly proportioned (range 0–1; see Appendix A for an example of the task).

The third-order false-belief task (FBT3) follows the classical structure of first- and second-order false-belief tasks, in which subjects have to attribute a cause to a character's behaviour on the basis of her/his mental state. The FBT3 consists of a brief story about three brothers involved in an unexpected transfer, followed by a questionnaire pertaining to the characters’ mental states. The questionnaire consists of one second-order false-belief question and two third-order false-belief questions (closed-ended questions), each followed by a respective justification question (open-ended questions). All false-belief answers are scored as either correct (1) or incorrect (0); for the justification questions, a 0 is given for incorrect answers, a 1 is assigned when the answer concerns a behaviour of the character, and a 2 is given when the answer concerns the characters’ mental state. The range of the FBT3 total score was proportioned and was from 0 to 1.50 (see Appendix B for the complete versions of the task).

#### Working Memory and Executive Function Tasks

Working memory was assessed through the Listening Span Test (Daneman & Carpenter, 1980; Pazzaglia, Palladino, & De Beni, 2000), which consists of two groups of sentences. Each group is composed of a sequence of sentences increasing from two to six; the sentences are tape-recorded and listened to by the subjects. At the end of each sentence, the subject must decide if it is true or false; this additional task was included to control for the fact that participants may have just concentrated on the final words rather than processing the entire sentence. Each sequence of sentences is presented in ascending order, starting with the two-sentence sequence and continuing to the six-sentence sequence; at the end of each sequence, the subject must recall the last word of each sentence. The score is based on the total number of words correctly recalled in the correct order and ranges from 0–40.

Executive function was assessed by the Clock test (Fabio, Antonietti, & Pravettoni, 2008; Moron, 1997, as cited in Fabio et al., 2008). This task evaluates the access to automation and the return to voluntary control through the presentation of four tables, each filled with 400 watches (among which there are 40 targets). In the first, second, and third table, the subject has to identify all the clocks showing 04:00, which activates automation processes. In the last table, the participant has to identify the clocks that show 05:00, which requires a return to voluntary control. The time required to complete each table is three minutes.

We calculated the following indices to evaluate the subjects' performance:

1. Inaccuracy index: the sum of the number of incorrect answers and omissions (range 0–160)
2. Automation index: the number of correct answers in the third table minus the number of correct answers in the first table (range -40–40)
3. Rigidity index: the number of correct answers in the third table minus the number of correct answers in the fourth table (range -40–40)

#### Language Tasks

To control for verbal skills, we administered the Vocabulary and the Comprehension subscales of the WISC-III (under 16 years [Wechsler, 1991]) and the WAIS-R (over 16 years [Wechsler, 1982]). The Vocabulary subscale of the WISC-III and WAIS-R is composed of 30 and 35 words, respectively, that subjects must define. A score of 0 (incorrect answer), 1 (partially correct answer), or 2 (correct answer) is given for each definition, and total score is weighted according to the scoring manual. The total score ranges from 1–19. The Comprehension subscale is composed of 18 (for the adolescents) or 16 (for the adults) sentences that the subject is asked to explain. A score of 0 (incorrect answer), 1 (answer partially correct), or 2 (correct answer) is given for each answer, and total score is weighted according to the scoring manual (range 1–19).

Subjects completed the tasks, which were presented in the Italian, in a quiet room in the school or at the University. The tasks were completed in two group sessions: the Listening span test, IMT, and Vocabulary task were completed in the first session; and the Clock test, FBT3, and Comprehension task were completed in the second session.

## Results

Table 1 reports the descriptive statistics for the explored variables, namely the total scores of each task that were used in the subsequent analyses. First, a preliminary evaluation of subject performance on the two theory of mind tasks is presented. Second, the possible links between these tasks, as well as between theory of mind tasks and cognitive variables are explored. Finally, a general model that evaluates the impact of gender, age, and cognitive variables on theory of mind performance is presented.

**Table 1:** Descriptive Statistics of Task Performance

	Young adolescents (*N* = 47)				Adolescents (*N* = 43)				Young adults (*N* = 20)				Total sample (*N* = 110)			
Tasks (score range)	Min	Max	*M*	*SD*	Min	Max	*M*	*SD*	Min	Max	*M*	*SD*	Min	Max	*M*	*SD*
FBT3-lev2 (0/1.50)	.00	1.50	.78	.45	.00	1.50	.91	.44	.00	1.50	1.00	.40	.00	1.50	.87	.44
FBT3-lev3(0/1.50)	.00	1.50	.36	.47	.00	1.50	.29	.44	.00	1.50	.74	.66	.00	1.50	.40	.51
FBT3 (0/1.50)	.00	1.33	.50	.38	.00	1.33	.50	.34	.33	1.50	.82	.43	.00	1.50	.55	.39
IMT-lev1 (0/1)	.75	1.00	.98	.06	.75	1.00	.99	.05	1.00	1.00	1.00	.00	.75	1.00	.99	.05
IMT-lev2 (0/1)	.67	1.00	.94	.13	.67	1.00	.98	.07	1.00	1.00	1.00	.00	.67	1.00	.97	.10
IMT-lev3 (0/1)	.20	1.00	.79	.19	.40	1.00	.87	.14	.80	1.00	.89	.10	.20	1.00	.84	.17
IMT-lev4 (0/1)	.50	1.00	.88	.14	.50	1.00	.85	.16	.75	1.00	.87	.13	.50	1.00	.87	.15
IMT-lev5 (0/1)	.00	1.00	.85	.36	.00	1.00	.81	.39	.00	1.00	.95	.22	.00	1.00	.85	.35
IMT (0/1)	.71	1.00	.89	.08	.65	1.00	.91	.08	.82	1.00	.93	.05	.64	1.00	.90	.08
Listening span test	20.00	40.00	32.81	4.57	28.00	40.00	35.65	3.43	25.00	39.00	33.20	4.25	20.00	40.00	33.99	4.28
Inaccuracy (0/160)	17.00	118.00	54.42	23.46	7.00	77.00	39.28	14.64	13.00	82.00	46.55	18.02	7.00	118.00	47.07	20.46
Automation (-40 / 40)	-12.00	25.00	3.21	7.16	-9.00	15.00	4.02	6.13	-8.00	8.00	1.40	4.44	-12.00	25.00	3.20	6.36
Rigidity (-40/ 40)	-16.00	8.00	-2.64	5.15	-16.00	8.00	-2.02	4.64	-19.00	10.00	-.65	7.06	-19.00	10.00	-2.04	5.36
Vocabulary (1/19)	5.00	15.00	11.06	2.26	10.00	19.00	14.95	1.99	4.00	17.00	13.40	3.50	4.00	19.00	13.01	2.99
Comprehension (1/19)	1.00	17.00	10.62	2.98	9.00	17.00	13.65	2.00	9.00	17.00	12.40	2.50	1.00	17.00	12.13	2.88

A General Linear Model for repeated measures was employed to compare participants’ performance at the five levels of recursive thinking investigated by the IMT. The model reveals a main effect of the levels (*F*(4, 106) = 35.144 *p < .*001, η_p_^2^ = .570, θ = 1). More specifically, pairwise comparisons (Sidak correction) show that participants comprehend the first and second levels of recursive thinking (respectively *M* = .989, *M* = .970) significantly better than the third, fourth, and fifth levels of recursive thinking (respectively *M* = .840, *M* = .868, *M* = .855). Furthermore, regarding FBT3 performance, a paired sample *t*-test showed that second-order false belief is understood significantly better than third-order false belief (*t* = 7.741, *df* = 108, *p* < .001; respectively *M* = .872, *M* = .404).

With regard to the link between the two recursive thinking tasks, FBT3 performance and the third level of recursivity of the IMT are positively associated (*r* = .198, *p* = .039). However, this association disappears after controlling for working memory and language comprehension (*r* = .142, *p* = .143).

The links among recursive thinking abilities, working memory, language competency, and executive function in the entire sample were also investigated. Results show a significant correlation between total IMT score and working memory (*r* = .252, *p* = .008). Both the third-order false belief understanding (assessed through the FBT3) and the third level of recursivity of the IMT positively correlate with the Comprehension task (respectively, *r* = .233, *p* = .015; *r* = .314, *p* = .001).

Univariate ANOVAs revealed an age effect on the third level of recursivity and total IMT score (respectively, (*F*(2, 107) = 4.441 *p* < .05; *F*(2, 107) = 3.128 *p* < .05), as well as on the FBT3 (*F*(2, 106) = 5.862 *p* < .01; *F*(2, 106) = 6.157 *p* < .01). As for the third level of recursivity of the IMT, young adolescents’ performance (*M* = .787) was significantly worse than that of other participants (*M* = .874, *M* = .890); for IMT total score, the difference persisted only between young adolescents (*M* = .886) and young adults (*M* = .935). As for the third-order false belief of the FBT3, young adolescents (*M* = .364) and adolescents (*M* = .290) performed significantly worse than young adults (*M* = .737); this difference was also observed for FBT3 total score (*M* = .503 and *M* = .496 vs. *M* = .825).

**Figure 1:**
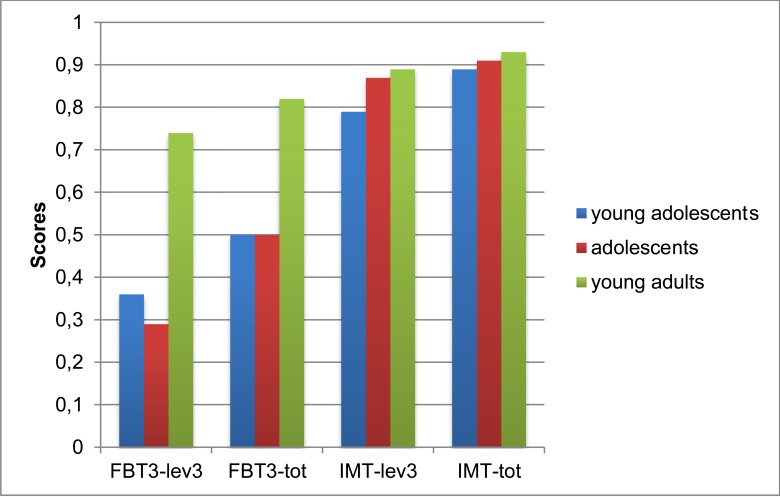
Age effect on third levels and total scores of Imposing Memory Task and False-Belief Task.

On the basis of these results, we constructed a multivariate general linear model in order to explore the effect of gender and age on the total score and the third level of recursivity of the IMT, as well as on FBT3 performance, controlling for working memory and comprehension. The model shows a main effect of age (*F*(6, 200) = 3.460, *p < .*01, η_p_^2^ = .094, θ = .942). More specifically, tests for Between-Subjects effects show that age differences only affect FBT3 performance (*F*(2, 101) = 6.712, *p < .*01, η_p_^2^ = .117, θ = .909): young adults (*M* = .810) perform significantly better than young adolescents (*M* = .557) and adolescents (*M* = .443) (see Figure 1).

## Discussion

The present research investigates recursive thinking ability in adolescence and in early adulthood. Furthermore, we collect some evidences as contribution to validation of a new third-order false-belief task. Our results suggest that the first and second levels of recursive thinking in the Imposing Memory Task are better understood than the other levels. Considering performance in the third-order false-belief task, second-order false belief is understood significantly better than third-order false belief. These data highlight the importance to study the third level of recursive thinking during adolescence and early adulthood. In fact, the lower levels are well understood in both tasks, while the third level seems to require a leap of reasoning that was reflected by the greater difficulty subjects experienced in providing correct answers.

Furthermore, performance on third-order false-belief task is associated with the third level of recursivity of the Imposing Memory Task. This result suggests that both tasks measure the same construct of theory of mind, operationalized in a different manner. The correlation between the performance on this level of recursive thinking and language comprehension seems to support the hypothesis that the third level of recursive thinking represents an important step in theory of mind development. Adolescents and adults exploit their language skills in order to respond specifically to this level. These skills seem unnecessary for the previous levels, which are well understood, and for the subsequent ones, which are probably so difficult that good language skills do not help.

A correlation between working memory and performance on the Imposing Memory Task was found. Two characteristics differentiate the Imposing Memory Task from the third-order false-belief task, and these may explain this result. First, the Imposing Memory Task demands that one keeps in mind the perspective of multiple characters involved in numerous situations, while the third-order false-belief task requires one to remember a single story with only three characters. Second, the Imposing Memory Task asks subjects to identify the correct answers among a set of alternative couples, which requires one to remember the perspective of multiple characters and make inferences afterwards about their thoughts. Conversely, the third-order false-belief task requires the subject to make only one inference about the behaviour of two characters. Thus, it can be assumed that when completing the Imposing Memory Task, subjects use working memory more than in the third-order false-belief task.

Results show an age effect on both the total score and the third level of recursive thinking for the Imposing Memory Task and the third-order false-belief task. The multivariate general linear model shows that after controlling for working memory and linguistic comprehension, the age effect persists only for third-order false-belief understanding, specifically that young adults perform significantly better than young adolescents and adolescents. Performance on the Imposing Memory Task remains unchanged. The already described difference of operationalization between the two tasks may explain this result. This difference concerns the type of cognitive activity required to process the recursive thinking involved in the two tasks: the Imposing Memory Task requires one to recognize different levels of recursive thinking, whereas the third-order false-belief task requires one to make a causal attribution regarding the character’s behaviour, that is to make behaviour prediction after attributing a mental state. The age effect on the Imposing Memory Task disappeared when general cognitive functions are controlled. This may be explained hypothesizing that the variability of the performance to this task across ages mainly depends on the general cognitive components of the task itself. On the contrary, the age effect on the third-order false-belief task persists despite controlling for general cognitive abilities. In other words, this last task seems able to detect in a more direct way the peculiarity of the meta-representational mechanisms implied in the attribution of mental state and in the prediction of behaviour on the basis of this attribution. This evidence is consistent with the hypothesis of an increasing dissociation between theory of mind and cognitive skills starting during adolescence that is characterized by a greater propensity to consider the perspective of others (Dumontheil et al., 2010). This dissociation becomes evident in early adulthood in the present study. It is conceivable that, beginning at this age, the accuracy in the application of recursive thinking does not depend on cognitive abilities, which have now reached a high level, but rather depends on the individual’s richer social experience and more sophisticated ability to verify inferences on the basis of behaviour. From this perspective, the classic false-belief tasks, considered as the core task to test theory of mind acquisition, but criticized and considered ineffective for studies with adults, may be re-evaluated (Birch & Bloom, 2004; Bloom & German, 2000). Our results show that this task may be able to better detect developmental differences in the application of recursive thinking towards behaviour prediction than the Imposing Memory Task. The latter seems to be more strictly linked to the general cognitive demand, although it explores a wider range of levels of recursivity.

Several limitations of the present study warrant consideration. First, given the importance of the third recursive level of thought, the use of only one specific task (the third-order false-belief task) may have limited the validity of the results obtained, although the correlation between the two tasks used provides support to the commonality of the construct measured. In the future, it will be important to use a greater number of tasks to assess third-order recursive thinking. Furthermore, although the tasks used here propose daily life situations, it would be interesting to apply tasks with higher ecological validity with regard to both content and answer mode (for example, the Imposing Memory Task imposes reasoning on a multiple-choice response platform, which is generally not the case).

In conclusion, we believe that our results highlight two key points: the first concerns the possibility that the third level of recursive thinking represents an important step in theory of mind development from adolescence to adulthood; and the second concerns the revaluation of the classical false belief tasks, since they are able to capture some aspects of the application of theory of mind in adulthood that might not otherwise emerge. Future research will further test whether the false belief task, alongside the most advanced and recent tasks, could still provide useful evidence about the use of theory of mind within a life-span perspective.

## Appendices

### Appendix A: Imposing Memory Task: example ‘Where’s the post office?’

Taken from Kindermann et al., 1998, Appendix

Sam wanted to find a Post Office so he could buy a Tax Disc for his car. He asked Henry if he could tell him where to get one. Henry told him that he thought there was a Post Office in Elm Street. When Sam got to Elm Street, he found it was closed. A notice on the door said that he had moved to new premises in Bold Street. So Sam went to Bold Street and found the new Post Office. When he got to the counter, he discovered that he had left his MOT certificate at home. He realized that, without an MOT certificate, he could not get a Tax Disc, so he went home empty-handed.

Following the story, subjects were presented with questions similar to the example below (the score associated with each response option is shown in parentheses):

- Henry thought Sam would find the Post Office in Elm Street (1)
- Henry thought Sam would find the Post Office in Bold Street (0)

### Appendix B: Third-order false-belief task

Mark, James, and Luke are three brothers.

Mark is playing with a ball in the bedroom while his brother James is playing on the computer. After the game, Mark decides to go to the kitchen to eat a snack and puts the ball in a closed box. James and Luke see Mark put the ball in the box. While Mark is in the kitchen, James and Luke enter the bedroom. James takes the ball to play, puts it in the closet, and returns to the computer. At this point, Luke takes the ball and plays with it. James goes to the bathroom. Meanwhile, Luke hits the ball with his foot awkwardly, and the ball drops behind a large wardrobe. Luke is unable to recover the ball, so he climbs onto the bed and starts reading. James meets Mark in the hallway and tells him that he put the ball in the wardrobe. After several minutes, the two brothers enter in the bedroom. James goes to the computer, and Luke makes an unequivocal gesture to tell Mark that the ball is behind the wardrobe. James does not see this gesture. Mark wants to play again with the ball.

The questionnaire was as follows (the score attributed to each closed-ended answer is shown in parentheses):

1. *2-order false-belief question*: Where does Luke think that James will seek the ball? (in the wardrobe = 1; other = 0)
2. *Justification question*: Why?
3. *3-order false-belief question:* Where does Luke think that James thinks that Mark will look for the ball? (in the box = 1; other = 0)
4. *Justification question:* Why?
5. *3-order false-belief question*: Where does James think that Luke thinks that Mark will look for the ball? (in the box = 1; other = 0)
6. *Justification question:* Why?
